# Understanding the corporate political activity of the ultra - processed food industry in East Asia: a Philippines case study

**DOI:** 10.1186/s12992-023-00916-x

**Published:** 2023-03-06

**Authors:** Oliver Huse, Erica Reeve, Paul Zambrano, Colin Bell, Anna Peeters, Gary Sacks, Phillip Baker, Kathryn Backholer

**Affiliations:** 1 Global Centre for Preventative Health and Nutrition, Faculty of Health, Institute for Health Transformation, Deakin University, Geelong, Australia; 2 Alive & Thrive Southeast Asia, FHI 360, Manila, Philippines; 3 Faculty of Health, Institute for Physical Activity and Nutrition, Deakin University, Geelong, Australia

**Keywords:** Nutrition, Health policy, Corporate political activity, Ultra - processed foods, Philippines

## Abstract

**Background:**

Evidence is mounting that the ultra - processed food industry seeks to influence food and nutrition policies in ways that support market growth and protect against regulatory threats, often at the expense of public health. However, few studies have explored how this occurs in lower - middle income countries. We aimed to explore if and how the ultra - processed food industry seeks to influence food- and nutrition - related policy processes in the Philippines, a lower - middle income country in East Asia.

**Methods:**

Semi - structured key informant interviews were conducted with ten representatives from the Philippines government and non - government organisations closely involved with nutrition policy making in the Philippines. Interview schedules and data analysis were guided by the policy dystopia model, which we used to identify the instrumental and discursive strategies used by corporate actors to influence policy outcomes.

**Results:**

Informants were of the view that ultra - processed food companies in the Philippines sought to delay, prevent, water - down and circumvent implementation of globally recommended food and nutrition policies by engaging in a range of strategies. Discursive strategies included various tactics in which globally recommended policies were framed as being ineffective or highlighting potential unintended negative impacts. Instrumental strategies included: directly engaging with policymakers; promoting policies, such as industry - led codes and practices, as substitutes for mandatory regulations; presenting evidence and data that industry has generated themselves; and offering gifts and financial incentives to government individuals and agencies.

**Conclusions:**

In the Philippines, the ultra - processed food industry engaged in overt activities designed to influence food and nutrition policy processes in their favour. A range of measures to minimise industry influence on policy processes should be introduced, to ensure that implemented food and nutrition policies align with best practice recommendations.

**Supplementary Information:**

The online version contains supplementary material available at 10.1186/s12992-023-00916-x.

## Background

Countries in East Asia are facing an increased burden of diet related noncommunicable diseases (NCDs), including type 2 diabetes [1–5], liver disease [2, 6] and cardiovascular disease [2–5, 7], amongst other diet - related diseases [2, 4, 5]. This is being fuelled by a shift in diets away from traditional foods, and meal preparations and cuisines made from these foods, towards a greater consumption of animal - sourced and in particular ultra - processed foods and beverages (UPFs) [3, 5, 8–11]. UPFs are formulations of cheap, industrial ingredients, typically created by a series of industrial techniques and processes [12]. They typically contain little - to-no whole foods, are ready - to -eat or heat, are high in fat, salt and / or sugar, and low in fibre and protein. Strong evidence links dietary exposure to UPFs with higher risks of all - cause mortality, obesity, cardio - metabolic diseases, cancer, gastro - intestinal disorders, and depression, among others [13–17]. Increased consumption of UPFs has been associated with increased greenhouse gas emissions and pollution, with significant implications for the development of sustainable food systems [18–21]. Although the World Health Organization (WHO) and public health practitioners are encouraging governments in the East Asia region (and elsewhere) to introduce policies to protect against dietary harms and improve population diets, the UPF industry stands in opposition to these goals.

The UPF industry influences food and nutrition policies in ways that support market growth and protect against regulatory threats, often at the expense of public health. Many studies report significant increases in the market and political power of transnational food corporations in a globalized economy [22, 23], and with this have come concerns about their undue influence over food and nutrition governance and policy processes [24–29]. Corporate political activities (CPA) seek to defeat, delay, weaken, circumvent and/ or overturn proposed and implemented food and nutrition policies [30]. To achieve these objectives the UPF industry has been observed to use a wide range of strategies, which are consistently adopted globally [24, 26–29], even in small island states like Fiji [25]. Industry’s building of coalitions and framing of arguments are both frequent and consistent across countries, though may also be tailored to the local context [24–27, 29]. However, few studies have sought to understand how the UPF industry influences food and nutrition policy processes in lower - middle income countries (LMICs), or in Asia, and it is likely that contextual differences influence the strategies and tactics adopted by industry.

The Philippines is a LMIC in the East Asia region [31] with a rising per capita UPF consumption [8, 9, 11]. Since 2012 the government of the Philippines has introduced a range of policies to protect against dietary harms and malnutrition, including a tax on sweetened beverages, restrictions on unhealthy food and beverage marketing in schools, a voluntary ‘healthier choices’ logo to identify healthier products, guidelines on food provision in schools, and regulation of the marketing of breastmilk substitutes [11, 32–35]. The Philippines is a good case study for the actions of UPF corporations in other LMICs, as the government has sought to adopt a range of policies to address the heavy and rising double burden of malnutrition [11, 32–35], and there is some evidence the UPF industry has sought to avoid and undermine these policies [32–35]. Along with other LMICs in the region the Philippines is being targeted as a growth market for the food industry [11], but they face a number of challenges in mitigating this expansion and protecting the policy space [36].

This study aimed to explore if and how the UPF industry seeks to influence food- and nutrition - related policy processes in the Philippines. Findings may aid in the identification of strategies to reduce corporate influence on food and nutrition policy making, in the Philippines and in other LMICs in the East Asia region and globally.

## Methods

### Conceptual frameworks

We used a qualitative thematic analysis study design [37], using key informant interviews, to understand actions from the UPF industry that may influence food and nutrition policy processes in the Philippines.

While other frameworks have been suggested for analysing corporate influences over policy processes [38, 39], these have been criticised for over emphasizing the potential role of industry in policy processes and failing to capture corporate motivations [30]. We aimed to take a more critical approach to understanding not only how the food and beverage industry engages in policy processes in the Philippines but also why industry does so. We also aimed to better understand the nature of industry voices in policy discourse. Accordingly, data collection and analysis methods were guided by the policy dystopia model (Table 1) [30, 40]. While this model was developed for an analysis of the tobacco industry [30], prior research suggests that it is also appropriate when it comes to analysis of political activities of the food and beverage industry [40]. The policy dystopia model outlines two discursive and five instrumental strategies used by corporations to influence policy process [30, 40]. Discursive strategies are the argument - based strategies, or the ‘dystopic narratives’ by which industry frames policies as dystopic or dysfunctional [30, 40]. These discursive strategies are similar across different food and nutrition policy proposals and broadly fall into two categories; arguments that expand policy costs, and arguments that deny policy benefits. Instrumental strategies are the action - based strategies and techniques that corporations use to communicate their dystopic narratives [30, 40]. We did not include one instrumental strategy from the original policy dystopia model, illicit trade [30], as it is outside the scope of food and beverage corporate activity [40]. The policy dystopia model also outlines the range of industry - preferred outcomes that eliminate or limit the likely impact of policy on corporate profits [30, 40].

**Table 1:** Policy dystopia model

Theme	Sub - theme	Practices and descriptions
Discursive strategies	Expand policy costs	Unanticipated costs to the economy and society Unintended benefits to undeserving groups Unintended costs to public health
	Deny policy benefits	Deny intended public health benefits Argue costs to targeted industry
Instrumental strategies	Coalition management	Establish relationships with key opinion leaders and health organizations Seek involvement in the community Establish relationships with the media Constituency fabrication Opposition fragmentation and destabilisation
	Information and messaging	Production of information Amplification of supportive evidence Suppression of opposing evidence Presenting information in a credible manner
	Direct involvement and influence in policy	Indirect access to policymakers Offering of incentives Making of threats Actor in government decision making
	Legal actions	Use legal action (or threat of) in opposition to pro - health actors Influence the development of trade and investment agreements
	Illicit trade	Facilitating or conducting of smuggling
Corporate motivations	Weakening of the policy	Corporate lobbying aiming to weaken a proposed policy.
	Delay of the policy	Corporate lobbying aiming to delay a proposed policy.
	Defeat of the policy	Corporate lobbying aiming to prevent the implementation of a proposed policy.
	Avoiding or circumventing the policy	Changes to corporate policy or products to circumvent introduced policies.
	Overturning the policy (rear - facing strategy)	Corporate lobbying aiming to remove an implemented policy.
	Foreclosing (pre - empting) the policy (future - facing strategy)	Corporate lobbying aiming to prevent the proposition of a policy in the future.

### Context

The Philippines is a LMIC in the East Asia region [31]. The Philippines has adopted a presidential democratic constitutional republic system of government. This includes two legislative branches of congress: the Senate and the House of Representatives; a Judiciary, with the Supreme Court as the highest body; and an Executive, including the President and Cabinet [41]. Lawmaking requires a draft bill to be submitted to, and approved by, both the Senate and the House of Representatives before final approval or vetoing by the President. An example of such a bill is the sweetened beverage taxation policy included as part of the *Tax Reform for Acceleration and Inclusion Law* [32]. The executive branch of government (including the Departments of Health, Education, Finance, and others) may publish policy orders within the fields that they have a mandate for. An example of such an order is the ‘*Policy and Guidelines on Healthy Food and Beverage Choices in Schools and DepEd Offices*’, released by the Department of Education [35]. The President may also issue executive orders, which have the force and effect of laws. One such executive order is the ‘Philippines Milk Code (Executive Order 51)’ [34].

Government is strongly decentralized, with semi - autonomous Local Government Units interpreting and enforcing laws and policies.

### Participant recruitment

Twenty - two interviewees were purposefully sampled by Philippines - based research team members as having had been directly involved, or had experiences with, the development and implementation of food and nutrition policies in the Philippines. We chose to confine our recruitment sample size to 22 key individuals as they were recognised by in - country contacts as being most relevant to our topic of interest. Further, qualitative studies aim to obtain rich information over representative results, and interviews should stop when ‘information redundancy is reached’ [42]. As we were conducting an in - depth study with policymakers and other key stakeholders, we were not seeking representation in our sample, and instead felt that a more exclusive number of expert participants would provide greater depth than a larger number of participants from a more diverse range of backgrounds.

We targeted both houses of the legislative branch of the Philippines Government (the House of Representatives and the Senate), the Philippines Department of Health and the Department of Education, United Nations (UN) agencies, and nutrition - focused non - government organisations (NGOs). Industry groups were not approached to participate as we were interested in the perceptions of industry’s discourse and activities according to those closely involved in the policymaking process, rather than industry’s own reporting of these activities. Further, we were interested in speaking to stakeholders who identified food and nutrition policies as a priority, and prior research has shown that industry more often than not has a negative view of food and nutrition policy making [43]. Snowball sampling was used to recruit further interviewees [44].

Of twenty - two interview invitations, ten interviewees participated in our study, representing. a range of government agencies (*n* = 7) and public interest NGOs (*n* = 3). Government agencies included both the legislative and executive branches of the Philippines government, and NGOs included both Philippines - based health and nutrition NGOs, and UN agencies. In the case of two participants, technological issues made interviews difficult to facilitate, therefore written responses were provided to open - ended interview questions. The remaining eight interviews were conducted virtually using video conferencing software, with interviews lasting between 40 and 75 minutes.

Ethical approval to conduct this research was granted by Deakin University’s Human Ethics Advisory Group – Health, and the National Ethics Committee of the Philippines. All interviewees provided written informed consent to participate.

### Data collection

All interviews were semi - structured, guided by an interview schedule (Appendix 1). The interview schedule was primarily informed by the policy dystopia model (Table 1) [30, 40], but was also influenced by the literature relating to corporate power [22, 23] and countering corporate influence over policy processes [43, 45–47]. All interviews were conducted by one researcher, and (due to COVID - 19 restrictions) were conducted virtually.

### Data analysis

All interviews were transcribed verbatim and thematically analysed [48]. This included assigning descriptive labels to data, which were then grouped into categories. All coding was facilitated by the qualitative data management software NVivo 12 [49]. Deductive coding, according to the policy dystopia model (Table 1) [30, 40], was then used to group categories into key themes that described the activities that the UPF industry engage in to influence food and nutrition policy processes in the Philippines. We inductively added a code to collate data on countering corporate influence over policy processes [43, 45–47]. One author (OH) analysed all interview transcripts, with a second author (ER) analyzing a subset of interview transcripts (*n* = 3) to validate the analytic framework and assist with synthesizing key themes. Themes were narratively described and synthesised [50].

## Results

### UPF industry strategies to influence food and nutrition policy processes

Key themes and subthemes related to UPF industry strategies to exert power over and influence food and nutrition policy processes in the Philippines are summarised in Table 2 and described in detail below.

**Table 2:** Key themes describing the power and influence of the UPF industry in the Philippines

Subtheme	Description	Descriptive quote
***Corporate objectives***		
Delay policy implementation	Industry aims to delay policy implementation.	*“So that’s really that’s my observation, that it’s not always a proactive support, that there’s always that “we’re not ready, can you delay this?”***Interviewee 1**
Prevent policy implementation	Industry aims to defeat policy implementation.	*“The debate happened, and it did not prosper because at an early stage, as we say in policy, people* [opposers of the bill] *started throwing rocks at the idea. So people* [supporters the bill] *said this is not going to fly, so let’s just keep it under wraps. For now, let’s focus on something else.”***Interviewee 4**
Weaken proposed policies	Industry aims to weaken proposed policies by changing definitions.	*“For example, they were trying to lower thresholds so that certain foods from the industry would be able to be mass marketed*.” **Interviewee 8**
Circumvent implemented policies	Industry aims to use its power and influence to circumvent compliance with implemented policies.	*“They are unsupportive of the policies because they still sell and promote their products [in schools] even though we have policies in place.” ***Interviewee 6**
Amend implemented policies	Industry aims to amend policies that have already been implemented.	“*They tried to lobby for the amendment of the Philippines Milk Code, and a proposed bill* [which was supported by industry] *actually tries to water down the existing milk code.*” **Interviewee 9**
***Instrumental strategies***		
Coalition management	Industry actors form coalitions to amplify their messages and increase the combined power that they hold.	*“Forming a coalition of, you know, coconut oil supporters and having such power and visibility during policy discussions that really affect how the bill, or the proposed legislation goes*.” **Interviewee 3**
	Industry engages in marketing, sponsorship and corporate social responsibility activities to sway legislators’ constituents to their side.	*“You don’t want to be the bad guy* [make unpopular decisions] *in policy discussion, and if they* [industry] *are perceived as the good guy then well good luck to the government for pushing reforms.”***Interviewee 3**
	Industry sponsors government agencies who are subsequently beholden to them.	*“You can already see that there’s a conflict of interest when that’s the effect of getting the sponsorship, they* [industry] *sort of have an obligation for them to pacify them… allowing them to have a broader say in the development of the* [food and nutrition] *standards.”***Interviewee 8**
	Industry conducts research into key stakeholders to assist with directly engaging with them.	*“They do have committees or even like task groups assigned to fill up dossiers. Look at the backgrounds of people, who are in Congress, in the upper and lower houses… And then they deploy interventions that may be appropriate* [based on the information sourced in the dossiers]*.”***Interviewee 4**
Information management	Industry presents evidence, which commonly appears to come from highly reputable sources, that is interpreted to support industry arguments.	“*They’re going to use government research data to favour them or so that’s the puts the Research Institute in a quandary because the data is coming from a government agency. And it’s a matter of how they’re interpreting the data to favour them.*” **Interviewee 1**
Direct involvement and influence in policy	Industry engages in policy making processes, including consultation hearings and policy workshops.	*“When they* [the UPF industry] *are invited, they will of course send representatives. I’ve never heard of the UPF industry turning down these invitations. They will always show up. These are committee hearings. They will show up. They will have a formal position, papers they will submit.”***Interviewee 4**
	Industry promotes preferred alternative policies that are less likely to reduce sales of their products.	*If we push for a traffic light front - of-pack labelling scheme, it was very clear during our consultation that they’re pushing for a monochromatic scheme, that we know is not that effective compared to other schemes*. **Interviewee 3**
	Industry contacts policymakers directly, commonly by writing directly, invitations to industry - sponsored events, and engaging them in private meetings.	*“It may be in the form of a dinner meeting or lunch meeting, that they’re going to sit down with a legislator for them to discuss their agenda*.*”***Interviewee 1**
	Industry offers government agencies resources that they otherwise lack and implies the offer of gifts and similar incentives to individual policymakers.	*“They did not really offer us money or anything that vulgar, but they gave this impression that we can make your life easier, whatever you like, we’re going to give it to you.”***Interviewee 2**
Legal actions	Industry challenges, or threatens to challenge, policies through legal means.	*“The Association of the Beverage, the beverage industry, wrote to the Department of Education saying that the Department of Education does not have the mandate to formulate such a nutrition policy.”***Interviewee 8**
***Discursive strategies***		
Expand policy costs	Proposed policies will have negative impacts on the economy, by harming suppliers of raw ingredients and small businesses, and reducing government revenue.	*Especially in the context of the COVID pandemic, economic recovery is very much highlighted in the discussion so any proposal or legislation that will affect businesses, will probably have some negative reception*. **Interviewee 3**
	Policies will place a greater burden on low - income populations.	*“The sweetened beverage industry tried to get the sympathy of the public and the legislators’ support by saying that the taxation of sweetened beverages would result to massive lay - off of workers, hence is anti - poor.”***Interviewee 7**
Deny policy benefits	Proposed policies will not be effective at improving health.	“*I think two years ago, there was really a strong manifestation of the industry through the media expressing… how this legislative proposal will not help the health sector.*” **Interviewee 3**
	Industry products are not responsible for ill - health.	“*Their main argument was that consuming sugar sweetened beverages does not contribute to obesity*.” **Interviewee 2**
	Under, rather than overnutrition, is the primary health concern in the Philippines	*“Those perceptions of their healthfulness weren’t fully there yet because a large focus in the Philippines was still more on the undernutrition side… That notion was still there, and the companies really capitalized on that*.*”***Interviewee 5**
Industry as a policy expert	Industry positions itself as an expert that should contribute to policy discussions	[Industry] *are essentially viewed as a technical expert on the food system because they pretty much have the visibility in the food supply chain.***Interviewee 3**
***Countering corporate influence***		
Increased transparency and declarations	Recognition of the importance of increasing transparency and declaration of conflicts of interest regarding industry influence and engagement.	*“Working against corruption, working for transparency and disclosure of discussions with any representative of industry, no matter at what at what level, I think is extremely important, and not being intimidated by the arguments provided by industry but ensuring that policy making is in the best interests of the population.”***Interviewee 5**
Role of other actors	Non - government organisations and research - based actors both have a role to play in countering the political influence of the UPF industry.	“*I think if it* [food and nutrition policy] *was supported by* [more and stronger] *research, the food and beverage industry could not do anything to influence policy making*. **Interviewee 6**
Controlled industry engagement	Industry will continue to play a role in influencing nutrition policy in the Philippines and so it is important to identify key policy levers and protect them from industry influence.	*“If I was in a position of regulation, I would say that I am clear on some parts like example sodium. The level has to be this level. It’s a non - negotiable, but I’m willing to work with you on the alternatives.”***Interviewee 4**

All interviewees spoke about a range of policies which they had experience in, and the interviews were not restricted to specific policy types. Discussed policies included sweetened beverage taxation (2018) and salt taxation (not passed), the ‘Philippines Milk Code’ (1986), guidelines on unhealthy food and beverage availability and marketing in schools (2017), and restrictions on trans - fatty acid content within foods (proposed).

### Corporate goals and objectives

All informants reported the UPF industry in the Philippines as having engaged in a range of instrumental and discursive strategies to defeat, delay, weaken, circumvent and/ or overturn food and nutrition policies.

With regards to defeat, the UPF industry was reportedly able to defeat the introduction of a salt tax in the Philippines in 2019, during very early debates in the House of Representatives. Interviewees identified industry’s discursive messaging as a key factor that contributed to the failure of this bill to pass through parliament. When defeat was not possible and policies were adopted, interviewees recognised that industry often aimed to delay policy implementation:

*…it's not always proactive support… there's always that “we're not ready, can you delay this?”*. Interviewee 1

According to one informant, this included an attempt to delay implementation of the salt tax, giving industry time to formulate and propose their own alternative approaches.

Another perceived goal of the UPF industry was to weaken the design of proposed policies, for example, by increasing the number of products that were excluded from regulation. It was reported that this was the case for the sweetened beverage taxation policy, where lobbyists attempted to have certain beverage types exempted from the tax, and the restrictions on healthy food and beverage marketing in schools:

*For example, they were trying to lower thresholds so that certain foods from the industry would be able to be mass marketed*. Interviewee 8

Four informants also reported that the UPF industry attempting to circumvent or avoid policy implementation. For example, that the UPF industry continues to sell and promote unhealthy products in schools, despite a Department of Education order recommending against this:

*They are unsupportive of the policies because they still sell and promote their products [in schools] even though we have policies in place. *Interviewee 6

The UPF industry also reportedly attempted to overturn existing policies, sometimes up to 10 years after a policy had been introduced. Interviewees described how industry complied with a policy for a period of time but continued to engage in activities to either amend or remove policy. For example, industry saw a routine, government - led update of the Philippines Milk Code as an opportunity to propose their own changes to the law which would likely weaken the intended effects of the policy.

### Discursive strategies

Interviewees described how the UPF industry relied on consistent discursive strategies to counter pro - health policy frames. Discursive strategies are the argument - based strategies that industry uses to frame food and nutrition policies as dysfunctional [30, 40]. We also identified one novel discursive strategy not previously included in the policy dystopia model: a meta - framing adopted by industry to use policy consultations to frame itself as an ‘expert’ policy contributor.

#### Expanding policy costs

Interviewees described multiple cases where the UPF industry promoted a dystopic narrative arguing that a policy would harm the economy by harming local producers, increase unemployment and reduce government revenues. Notably, it was perceived that more recently industry had been drawing on the economic impacts of the COVID - 19 pandemic and the need for economic recovery as an argument against the introduction of food and nutrition policies:

*Especially in the context of the COVID pandemic, economic recovery is very much highlighted in the discussion so any proposal or legislation that will affect businesses, will probably have some negative reception*. Interviewee 3

Interviewees also described how industry has argued that a policy would harm low - income groups disproportionately and in the context of fiscal policies, would increase the prices of the foods they consume. As interviewees noted, industry had capitalised on this to directly counter discussions concerning both sweetened beverage and salt taxation policies in the Philippines:

*The sweetened beverage industry tried to get the sympathy of the public and the legislators’ support by saying that the taxation of sweetened beverages would result in massive lay - off of workers, hence is anti - poor*. Interviewee 7

#### Deny policy benefits

Another dystopic narrative adopted by industry commonly disputed the necessity and effectiveness of proposed policies. When disputing the effectiveness of a policy, industry often relied on rhetoric that the policy would have no impact on health outcomes and that their products were not harmful to health. For example, in response to discussions on a sweetened beverage tax in the Philippines the beverage industry argued that sugar - sweetened beverages do not contribute to overweight and obesity. It was also reported that industry capitalised on the long - term perception that undernutrition is the predominant health concern in the Philippines. In doing so, industry commonly adopted the position that their products were beneficial to health as they are calorie - dense and should be promoted for consumption.

*Those perceptions of their healthfulness weren't fully there yet because a large focus in the Philippines was still more on the undernutrition side… That notion was still there, and the companies really capitalized on that*. Interviewee 5

#### Industry as a policy expert

Another perceived UPF industry dystopic narrative was a ‘meta - framing’ related to industry positioning itself as a food and nutrition ‘expert’ in many policy making discussions. Interviewees described how the UPF industry had used availability meetings and consultations to successfully propagate a discourse establishing itself as an ‘expert’ in the process of developing food and nutrition policies. This framing by industry had been successful, as interviewees described the UPF industry’s visibility in policy discussions and acknowledgement by policymakers as key contributors. This in turn allowed industry greater avenues for disseminating their dystopic narratives. This was perceived as a considerable source of corporate power for industry.

[Industry] *are essentially viewed as a technical expert on the food system because they pretty much have the visibility in the food supply chain*. Interviewee 3

### Instrumental strategies

The UPF industry engaged in a range of instrumental strategies (action - based strategies) to communicate their dystopic narratives to policymakers and other key stakeholders.

#### Coalition management

Participants perceived coalition management as an industry tactic to amplify voices from the UPF industry, raise industry presence, and amplify the power of industry to influence policy processes. It also included actions to sway non - industry actors to industry’s side. Such coalitions reportedly included a range of industry associated actors, including large corporations and manufacturers, but also upstream and downstream actors, such as suppliers of raw ingredients and food and beverage retail groups. For example, one interviewee recalled that, in response to the proposition of legislation limiting trans fatty acid content of foods, the coconut manufacturers had engaged multiple actors associated with the coconut oil industry to oppose the proposed policy. This coalition was reported to include multiple industry actors, recruited through their shared interest in the industry, but was also reported to include academic actors who were reportedly offered financial incentives to speak on industry’s behalf. Interviewees perceived that the forming of this coalition increased the power of the UPF industry and its supporting actors to oppose and ultimately influence the passage of this bill. As one interviewee noted:

*Forming a coalition of, you know, coconut oil supporters and having such power and visibility during policy discussions that really affect how the bill, or the proposed legislation goes*. Interviewee 3

Participants reported that food industry actors also established coalitions with consumer rights agencies to jointly oppose policy. For example, one interviewee described how industry groups launched joint press conferences alongside consumer advocacy groups in opposition to the proposition of a sweetened beverages tax and a tax on salt. The participant described the framing of this policy as key to building a coalition; it was perceived that by arguing that the tax would harm consumers, industry positioned consumer rights agencies as an ally in policy discussions.

*We also had to compete with media advocacy; they* [industry] *had joint press conferences with consumer advocacy groups. *Interviewee 10

Interviewees also perceived industry as building coalitions that were inclusive of policymakers. Participants perceived that forming coalitions with policymakers provided industry with the potential to directly influence policy proceedings. One interviewee described how industry representatives were seen to sit directly alongside policymakers who had aligned with them during policy processes and act to influence their decisions:

*It's not really a hidden thing. You can identify and you can tell which legislators are like the mouthpieces of our industry… You would see industry representatives over there* [sitting next to policymakers in Parliament]*. They're typing on their index cards and then they would hand the index card to the legislator… The fact that they are in the gallery, and they are handing over cue cards to legislators, they are semi - formally participating*. Interviewee 4

Participants indicated that food industry actors also acted to sway public opinion to favour industry:

*It's one thing to interact directly with the legislators, the policy makers. It's another thing to interact with their constituents. So this is where you will see fast food* [companies] *sponsoring sports affairs and then hanging their banners at the side of the pitch and in a soccer match.  *Interviewee 4

Participants perceived the UPF industry as influencing public option through market and non - market activities. Market activities included product and brand marketing and sponsorship activities to create favourable public opinion of their products and brands. Non - market activities included using corporate social responsibility activities that participants perceived were aimed at distracting attention away from the harmful impacts of their products. Such activities were noted to be enabled by the vast resources that the UPF industry can access. Interview participants indicated that as policymakers were unlikely to go against the views of their constituents, such activities gave corporations considerable power to influence policy development:

*You don’t want to be the bad guy* [make unpopular decisions] *in policy discussion, and if they* [industry] *are perceived as the good guy then well good luck to the government for pushing reforms. *Interviewee 3

Industry targeted their coalition management strategies at policymakers who were likely to be most receptive to them. One interviewee reported how industry would fund the creation of dossiers describing policymakers. The interviewee described how these dossiers would analyse policymakers’ political positions and identify specific industry - backed ‘interventions’, as decided by the information contained in the dossiers. The interviewee described how these ‘interventions’ would in fact be the activities or offers most likely to be effective at ensuring a policymaker’s support (a provided example included offering a funded conference at a resort to a policymaker who enjoyed holidays):

*They do have committees or even like task groups assigned to fill up dossiers. Look at the backgrounds of people, who are in Congress, in the upper and lower houses… And then they deploy interventions that may be appropriate* [based on the information sourced in the dossiers]*. *Interviewee 4

Industry also pre - emptively acted to draw potentially pro - health actors to their side. Interviewees described how industry may sponsor or financially support certain government agencies. Interview participants then described the perception that such ministries were beholden to their industry sponsors.

*You can already see that there's a conflict of interest when that's the effect of getting the sponsorship, they* [industry] *sort of have an obligation for them to pacify them… allowing them to have a broader say in the development of the* [food and nutrition] *standards. *Interviewee 8

Finally, where industry could not sway policymakers to their side, they sometimes instead resorted to personal attacks on politicians through the media. It was described how the initial proponent of a tax on salt was forced to withdraw the bill in response to media campaigns directly describing the policymaker as ‘anti - poor’:

*The author had been receiving a lot of criticism* [in the media]*, that's why they withdrew the bill before it reached any committee meeting; “how come you* [the author] *are putting more of a burden on poor people?” *Interviewee 10

#### Information management

The UPF industry was reported to use information management to disseminate their discursive narratives and oppose policy adoption and implementation. Interviewees described how the UPF industry would fund and deploy corporate research to influence expert and policy discourse. Interviewees described that the data industry used to support its policy stance was often perceived as highly reputable, commonly published in academic journals, was often sourced from government datasets, or was presented by well - known nutrition experts. However, interviewees also perceived that this data was commonly unreliable and inaccurate, lacking in disclosures related to funding sources, and was simply being used to sway decision - makers to industry’s side.

*[Industry] will have formal position papers they will submit, and if you look at their position papers, they look scientific… If you are a policymaker or the staff of a policymaker, typically they are the ones who will receive the papers, and then they will look at its nice glossy sheets of paper with a nice, international sounding ‘journal of nutrition in developing countries’* [example, not factual, journal name]*, and then look at this finding that sugar sweetened beverage taxes have not been effective. *Interviewee 4

Interviewees also discussed industry’s use of government data, reporting that this was commonly ‘cherry - picked’ to support industry’s arguments.

[Industry] *is going to use government research data to favour them so that puts the institute in a quandary, because the data is coming from a government agency, and it's a matter of how they're interpreting the data to favour them. *Interviewee 1

#### Direct involvement and influence in policy

The UPF industry was reported to engage in the policy making process through both formal and informal channels, and through these channels industry was reported to communicate dystopic narratives directly to policy makers (without communicating through media as with information management strategies). Formal channels, including public consultations and policy development workshops, are required as part of the lawmaking process and provide an opportunity for industry and their representatives to voice their opinions and positions relating to specific policies. Interviewees voiced the opinion that industry would always seize the opportunity to speak through formal channels:

*When they* [the UPF industry] *are invited, they will of course send representatives. I've never heard of the UPF industry turning down these invitations. They will always show up. These are committee hearings. They will show up. They will have a formal position, papers they will submit. *Interviewee 4

Such formal meetings reportedly provided the UPF industry with an opportunity to promote food and nutrition policies that were relatively weak in design, from a public health perspective, as viable alternatives to policies that may have originally been proposed. Examples provided by interviewees included industry’s preference for nutrition education over unhealthy food and beverage marketing restrictions, and preferencing front - of - pack nutrition labelling scheme designs that are likely to be less effective at informing healthier food choices:

*If we push for a traffic light front - of - pack labelling scheme, it was very clear during our consultation that they're pushing for a monochromatic scheme, that we know is not that effective compared to other schemes*. Interviewee 3

Interviewees also reported that industry representatives were often called on by policymakers to contribute to policy development through policy development workshops, and this was an opportunity for dystopic narratives to be inserted into the policymaking process. For example, when discussing the development of nutrient profiling models (an approach for classifying foods and beverages as healthy or unhealthy based on nutrient criteria) in the Philippines, one interviewee reported that:

*They* [the technical working group for the development of a nutrient profiling model for the Philippines] *included in their expert panel a member of the Philippine Chamber of Food Manufacturers, and I attended one of the expert consultations and it seems that they are leaning towards the recommendations of that member from the food industry. *Interviewee 8

Informal channels of policy engagement were more irregular and included those that are not required as part of the lawmaking process, such as meetings, and direct and private contact between legislators and industry representatives. Interviewees described how, as such informal lobbying may not have been reported, this could be difficult to police and might lead to conflicts of interest. For example, interviewees reported that UPF industry representatives had direct contact with those responsible for policy development in efforts to communicate dystopic narratives and influence their perspectives to be pro - industry. Direct contact included writing directly to them, inviting them to industry - sponsored events, or hosting them at private, one - on-one meetings:

*It may be in the form of a dinner meeting or lunch meeting, that they're going to sit down with a legislator for them to discuss their agenda.* Interviewee 1

Interviewees described overlap between the private and legislative sectors such that some policymakers also had financial interests in food and beverage corporations, and such policymakers were likely to be receptive to dystopic framings. One interviewee described an instance where a policymaker had contacted a government agency to influence policy despite their vested interest:

*There is even a congressman who wrote to us. They used their congressional letterhead, but they were representing their own corporation, their private firm. So it's not ethical. But they did it anyway. *Interviewee 2

Several informants also described how the UPF industry offered policymakers gifts or other financial incentives in return for their support. It was reported that at an institutional level, this included provision of resources that government agencies otherwise lack, such as through the provision of funds for the National Nutrition Survey. At an individual level, several interviewees reported that, despite laws to the contrary, gifts and similar offers were commonly offered to policymakers. Interviews perceived that such offerings were designed to sway decision makers or may have been offered as a reward to policymakers who aligned with industry’s views. One interviewee explained how an industry stakeholder had implied offers of gifts in exchange for political support:

*They did not really offer us money…but they gave this impression that we can make your life easier, whatever you like, we're going to give it to you. *Interviewee 2

Aside from offering direct gifts to policymakers, industry was also reported to have made donations, commonly in the form of food and beverage products. While such donations were ostensibly to support nutrition and, in particular, responses to natural disasters (including the COVID - 19 pandemic), interviewees perceived them as an activity to generate political influence:

*They will say that to some politicians that “We donate this and that, you owe us bigtime, prioritize us when making a policy”.* Interviewee 6

#### Legal strategies

Two participants identified instances where the UPF industry leveraged legal means to influence the policy process. One interviewee noted that free - trade agreements and World Trade Organization obligations were often referenced by industry as grounds to object to a policy. In another case, an interview participant described industry’s legal opposition to a Department of Education policy regulating school nutrition environments. Industry argued that the Department of Education did not have the legal mandate to regulate school food environments, though no formal legal challenge was issued.

*The Association of the Beverage, the beverage industry, wrote to the Department of Education saying that the Department of Education does not have the mandate to formulate such a nutrition policy.* Interviewee 8

### Countering food and beverage political influence

There was a perceived need to reduce the UPF industry influence over food and nutrition policy making processes in the Philippines.

#### Increased transparency and declarations

Some informants perceived that it was difficult to monitor conflicts of interest between policymakers and industry actors, including pre - existing relationships and ongoing discussions between industry and political actors. Increased transparency and declarations of such conflicts of interest was recognised as a necessity. In particular, this would identify which food and beverage corporations are most active in disseminating dystopic narratives, and the avenues through which the dystopic framings are communicated. This would allow such conflicts of interest to be identified and be better managed and mitigated:

*Working against corruption, working for transparency and disclosure of discussions with any representative of industry, no matter at what at what level, I think is extremely important.* Interviewee 5

#### Role of other actors

The role of non - government actors in countering industry’s dystopic narratives in the Philippines could be strengthened. International development agencies (including the WHO) and civil society organizations (including various NGOs) were identified by interviewees as important actors that could oppose industry by submitting their own position papers and presenting pro - health arguments in relation to proposed food policies. However, interviewees perceived that the power of these agencies to influence policy processes is primarily as nutrition and technical experts. As industry is also viewed by many as a nutrition expert, there was some perceived overlap between these opposing groups, lessening the power of such pro - health actors to oppose industry. Hence, it was perceived that researchers and academics have a role to play in ensuring pro - health policymakers and NGOs have the evidence required to oppose industry arguments.

Interviewees also identified that a key source of influence for the UPF industry was their ability to form coalitions and unite voices, and it was perceived that many pro - health actors were not as organised. Interviewees believed that a key role of non - government agencies, such as the WHO, is to coordinate and support pro - health coalitions to promote food and nutrition policies, so that pro - health voices are a united and coherent message.

#### Controlled industry engagement

Interviewees recognised that to exclude the UPF industry entirely from the food and nutrition policy process would neither be practical nor effective. Indeed, amongst many interviewees there seemed to be a perception that industry could play a role in supporting the development and implementation of effective and equitable nutrition policies in some cases. This was notably the case where product reformulation may be required (such as salt iodization or food fortification laws). It was recognised that industry engagement should be monitored and managed to limit their influence on the policy design and the process for policy adoption. Further, key aspects of policy design should be defined prior to industry engagement in the process, to ensure policies align closely with best practice:

*If I was in a position of regulation, I would say that I am clear on some parts like example sodium. The level* [of specific policy elements or classifications] *has to be this level. It's a non - negotiable, but I'm willing to work with you on the alternatives* [other aspects of the proposed policy]*. *Interviewee 4

This was coupled with a view shared by some interviewees that industry had the potential to positively influence food environments and policies and that industry was making some positive changes. Such positive changes mostly took the form of product reformulation with an aim to ostensibly make products healthier:

*On the other hand, we see* [specific corporation] *trying to be more responsible, trying to refine their food products… not all of them are bad. We can see that in that case, some of them are also trying to improve in terms of eventually having healthier food options*. Interviewee 2

## Discussion

Our interviews with stakeholders in the Philippines revealed that CPA strategies operationalised by the UPF industry to influence policy centre on engagement with legislators and consumer groups to refute policy benefits, promoting alternative policies that are likely to favour corporate interests, constructing alternative evidence that undermined policy benefits, and offering incentives to government agencies and individuals. Through these strategies, industry disseminated pervasive messaging that framed policies as ineffective or as having unintended negative impacts, and that cultivated a positive public image. Our findings suggest that these have been impactful in delaying, preventing, watering - down and circumventing food and nutrition policies in the Philippines.

The CPA of food and beverage corporations in the Philippines are consistent with those applied in other countries globally [24–29]. In East Asia, several studies have described the corporate activities of the baby food industry [34, 51]. However, we are aware of only one other study that has described the political activities of the broader UPF industry in East Asia, which focused on Thailand [29]. Most prominently, this included use of information and messaging to shape dietary and public health debates and engagement with policymakers and communities to build coalitions. Similar to our findings in the Philippines, legal strategies were less often observed to be used by the food and beverage in Thailand. However, the Thai study did not report on industry’s use of financial incentives and policy substitution strategies. In contrast, we have given evidence that the UPF industry offers financial incentives that are perceived as aiming to compromise support for nutrition policies that are critical to addressing inequities and influencing nutrition outcomes. Further, we report on industry’s promotion of alternative food and nutrition policies in the Philippines, which are perceived as being more forgiving of industry’s interests and more likely to support market growth.

In countries around the world, the UPF industry has been reported to engage with policymakers, draw on evidence, form coalitions, and propose alternative policies [24–29]. Whilst our findings are similar in this regard, we have revealed more overt strategies by the UPF industry in the Philippines. For example, the food industry’s use of financial incentives and gifts have been rarely reported in other contexts [25–29], though this may be due to a reluctance of participants to report on these activities, rather than their inexistence. Previous studies have reported that key - informants have been uncomfortable discussing such instances, though their likely impact on the policy process is undisputed [25, 29]. This study provides real - world examples of either implied or actual financial incentives being offered to policymakers and other key stakeholders. This included industry donations to support local communities, but more significantly extended to overt cases where policymakers were offered incentives, including monetary offerings and industry - supported holidays, though many reports of the latter were repeats of second - hand information. The perception of such conflicts of interest has the potential to compromise the implementation of food and nutrition policies that are vital for supporting the consumption of healthy diets. Likewise, other interview - based studies have struggled to identify examples of industry activities to attack or destabilize opposition [25–29]. We describe industry - led analysis of stakeholders in the Philippines policy environment, ostensibly to better target these pro - health actors and policymakers with ‘interventions’ to advance a pro - industry agenda. This finding has not been previously identified in the literature.

We found the UPF industry in the Philippines opposed food and nutrition policies by disputing policy benefits and arguing excessive costs to limit implementation. These same arguments have been used by the UPF industry globally [52–54]. Academics have a role in developing context specific evidence to counter these arguments. We found that the UPF industry is leveraging the COVID - 19 pandemic in the Philippines to counter food and nutrition policy, arguing the need for economic expansion and positioning themselves as a provider of essential food and nutrition services. We argue that the COVID - 19 pandemic instead emphasises the need for food and nutrition policies, to reduce diet - related diseases, many of which are key risk factors for adverse outcomes associated with COVID - 19 [55, 56]. We are not the first to report on industry and other coalitions capitalising on such events. Sabatier and Weible [57] describe how external events are a key influence on policy subsystems. In the Philippines, the baby food industry capitalised on COVID - 19 related concerns, marketing through health claims, misinformation, and public donations to drive products sales in response to the pandemic (and contravening existing legislation) [58]. We also describe industry’s discourse to position themselves as policy ‘experts’, which had not been previously identified. It is possible that this framing is more targeted to LMICs, where the political institutions to support food and nutrition policies may not be fully established [11, 59], providing industry with an opportunity to frame itself as the solution to such absences.

Our study is the first to explicitly report on the specific strategies by the UPF industry to influence policy processes and build political power at a country - level in the East Asia region, though previous studies have commented on industry’s influence over specific policies [32, 33, 35]. Our findings align with existing literature describing the sources of corporate power of the UPF industry, globally. Notably, Wood et al. [60] and Milsom et al. [61] have synthesised a range of different corporate power frameworks to describe the different mechanisms by which transnational corporations draw on instrumental, structural and discursive sources of corporate power.

Several of the mechanisms described in these frameworks align with the real - world examples reported herein. For example, both frameworks describe corporate use of financial resources as a means of exerting influence and shaping policy processes [60, 61]. We describe how the UPF industry leverages financial resources in the Philippines to fund, amongst other activities, extensive marketing campaigns to sway public opinion and the offering of financial incentives to policymakers. Likewise, the ideological positioning of industry is seen as a source of political influence [60, 61]. Here, we describe how UPF industry actors frequently hold influential positions and are seen by many as food and nutrition experts when it comes to policy development, providing industry with perceived legitimacy and influence. Finally, Wood et al. [60] discuss industry’s formation of coalitions which amplify pro - industry voices to levels that exceed pro - health actors, which was also reported herein. Countering the multiple mechanisms by which industry builds corporate power will be essential for addressing political influence of the UPF industry in the Philippines.

We have highlighted leverage points and vulnerabilities where we must actively manage corporate influence on policies critical for addressing all forms malnutrition [45–47]. Interviewees identified that greater management of conflicts of interest and increased transparency in interactions between policymakers and private enterprises could help to mitigate industry influence over food and nutrition policy. This aligns with existing literature, identifying increased transparency, management of conflicts of interest, monitoring of and education about corporate practices, and prohibition of interactions between policymakers and industry [45, 46]. Some informants reported a perception that the UPF industry has a role to play in policy development and implementation. Any framework for addressing UPF industry influence should ensure that such engagement is free from conflicts of interest and that any engagement with industry and other key stakeholders within the policy development process promotes, facilities and compliments policy measures [43, 47]. Addressing corporate power will also be key, as the expansive power of the UPF industry has been identified as a key barrier to addressing the influence of this industry [60, 61].

The strengths of our study include the range of different government and non - government agencies and groups in the Philippines that we interviewed, capturing the views of people from a range of different areas. Further, our interview schedule and analysis approach were guided by a well - established framework relating to corporate influences over policy and corporate power, the Policy Dystopia Model [30], ensuring that our data collection and analysis aligns with the published literature on this topic.

Limitations include the focus on just one country in the East Asia region. It is possible that UPF industry political activities in the Philippines do not reflect the activities being conducted elsewhere in East Asia. However, the consistency in industry activities with other regions globally [24–29, 52–54], indicates that it is likely that the UPF industry is conducting similar activities in other countries, especially LMICs, in East Asia. Second, given our sample strategy, interviewees who agreed to participate may have a greater interest in health promotion and have less favourable opinions of the UPF industry compared to other policymakers in the Philippines. Future research may wish to consult a broader group of stakeholders, and this broader group of stakeholders could include industry actors, who were not included in the present study. Third, we asked interviewees about industry influence more generally, and did not discuss how industry CPA influenced particular policies. As part of a broader analysis of the policy development and implementation process, in - depth case studies to understand how industry influenced specific policies may be warranted.

## Conclusions

The UPF industry in the Philippines engaged in highly overt activities designed to influence food and nutrition policy processes in their favour. To counter the rising prevalence of diet - related non - communicable diseases in the Philippines public health must be prioritised before the conflicting motives of the UPF industry. Strong conflict of interest policies to promote increased transparency in engagement with policy processes will be important.

## Supplementary Information

**Additional file 1.** Interview discussion guide.

## Data Availability

To preserve participant confidentiality, interview transcripts for this research are not publicly available.

## Abbreviations

CPA: Corporate political activity
LMIC: Lower - middle income country
NCD: Non - communicable disease
NGO: Non - government organisation
UN: United Nations
UPF: Ultra - processed food
WHO: World Health Organization
