# Supplementary material for: Understanding the corporate political activity of the ultra - processed food industry in East Asia: a Philippines case study
Source: Global Health. 2023 Mar 6;19:16. doi: 10.1186/s12992-023-00916-x (PMC9986662; doi:10.1186/s12992-023-00916-x)
Supplement: Supplementary file 1 — Additional file 1. Interview discussion guide. [file 12992_2023_916_MOESM1_ESM.docx]

**Food and beverage industry influence over the policy making process in the Philippines: Interview discussion guide**

**Introduction**

Before we begin, please allow me to say a bit about the research and the interview today. To give you an indication, the interview should last for no longer than 45 to 60 minutes. Does this fit into your schedule today? If you’d like me to make this a shorter interview, please feel free to let me know now.

You may have read the Plain Language Statement already, but just in case you haven’t please let me know and I can go over it with you?

As background, the aim of this research is to explore the role of food and beverage organisations in shaping the food and beverage environment of the Philippines. When I talk about the food and beverage environment of the Philippines, I am talking about the range of environmental, social and political aspects that influence people’s ability to healthy healthfully and avoid both under and over nutrition. I am particularly interested in how the food and beverage industry influences the implementation of policies to address overweight in the Philippines. This research will involve the conducting of interviews with key stakeholders in the Philippines, such as yourself. As someone who is very knowledgeable about this topic, I would like to ask you a number of related questions.

We are also conducting interviews with other stakeholders in the Philippines. The answers that you give will be combined with the responses from these other interviews to address our research question. Combining all the interviews and looking for similarities in the topics raised will ensure that interviewees cannot be identified. Once these interviews are finished, we will draft a number of academic journal articles on the findings and will be happy to share these with you if you wish.

(*If not already signed*) I also note that you haven’t returned the consent form to me yet. Are you able to sign and do this now? *If the answer is no*: In that case could you please provide verbal consent to participate in this interview (*Ask to turn on recorder now if this is the case*).

Do you have any questions before we begin?

Are you okay if I turn on the voice recorder now? *(If not already on)*

| **Background** |
| --- |
| Can you please tell me about your current or past positions and how they do or have related to the food and nutrition in the Philippines? |
| Can you please tell me about some of the activities that you and/or your organisation are a part of that may influence the food and nutrition environment in the Philippines? |
| Can you please tell me about how you perceive the food and beverage environment to be in the Philippines?   - How would you describe the current policy and regulatory situation for supporting healthy food and beverage consumption?   - What are some of the good aspects of the nutrition environment as it stands?   - What are some of the negative aspects of the nutrition environment as it stands? |

| **Interview questions** |
| --- |
| The following questions relate to the various actors who have power to shape the food and beverage environment in the Philippines. Individuals and organisations |
| - Who do you believe are the key actors involved in the development and implementation of food and nutrition policy in the Philippines? *Mention industry as a prompt if needed.* |
| Let’s talk more about the role of the food and beverage industry within the development and implementation of food and nutrition policy in the Philippines.   - Can you describe the role of the food and beverage industry in the development, adoption and implementation of nutrition policy, and industry’s engagement with the process? I’m particularly interested in industry’s role in policies aiming to address overweight.   1. What are some of the ways in which industry is supportive of the policy process?   2. What are some of the ways in which industry is unsupportive of the policy process? - What are some things that industry does to try and influence food and nutrition policy in the Philippines?   1. Consider actions to gain public favour   2. Consider breaking up or disempowering opposing groups   3. Consider offering alternative policies   4. Consider challenging policies through the law   5. Consider industry’s information and messaging,   6. Consider funding research in other areas   7. Consider financial incentives and gifts from industry - What impact do you think the food and beverage industry has on effectiveness of policy to achieve objective (this might include changes to proposed policy, or changes to implemented policy)? What do you think are industry’s intended impacts?   1. Weakening of the policy   2. Delay of the policy   3. Defeat of the policy   4. Avoiding or circumventing the policy   5. Overturning the policy (rear-facing strategy)   6. Foreclosing (pre-empting) the policy (future-facing strategy) - Can you think of a time or example where industry has directly approached someone who is involved in the policy process to influence the policy process? - Why do you think that the food and beverage industry has the ability to influence food and nutrition policy in the Philippines?   1. The ability of industry to exert influence through engagement with key political players (*e.g. Through funding*).   2. The ability of industry to exert influence through occupying key positions (such as board members and senior roles in education and healthcare).   3. The ability of industry to exert influence through the provision of key goods and services. - Do you think that corporate influences need to be addressed? Do you have any specific ideas on how to counteract the power of the food and beverage industry? - Do you think the food and beverage industry has a role as a positive actor in shaping healthy food environments in the Philippines? |
| - Let’s consider other key actors who may influence the development and implementation of food and nutrition policies in the Philippines. What are the roles and positions of these actors? (*Go through unmentioned actors from list below*)   1. Political parties and politicians   2. Government agencies   3. NGOs   4. Foreign governments   5. Academics and other researchers; - How do you think the ability of these actors to influence food and nutrition policy compares to the ability of the food and beverage industry to influence food and nutrition policy?   Again, the key elements of power are:   1. The ability of industry to exert power through engagement with key political players (*e.g. Through funding*). 2. The ability of industry to exert power through occupying key positions (such as board members and senior roles in education and healthcare). 3. The ability of industry to exert power through the provision of key goods and services.  - Now that we’ve spoken about a large range of actors in the food environment, can you describe any positive influences that these actors have when shaping the food and nutrition environment in the Philippines? - What can be done to increase the ability of these actors to influence food and nutrition policy in a positive way? |

| **Closing** |
| --- |
| Thank you for your time today.   - Is anything additional that you would like to add? - Is there anyone else that you might be able to help me contact, who would be a good candidate for another interview? |
| That’s all for now then. I will transcribe this interview and send you a copy of the transcription for your records and confirmation. I will compile your responses with the responses of other participants. The overall outcomes from these interviews will then be included in academic publications.   - Would you like to receive a copy of the results? - If necessary, would you be OK if I contacted you again with any additional questions? - Do you have any final questions? |
